# Impact of Albuminuria and Renal Dysfunction on the Anemia-Improving Effect of SGLT2 Inhibitors in Patients With Type 2 Diabetes: A Real-World Observational Study

**DOI:** 10.1155/jdr/5399360

**Published:** 2025-06-28

**Authors:** Ayami Kajiwara-Morita, Kentaro Oniki, Akira Yoshida, Noboru Kurinami, Tomoko Suzuki, Fumio Miyamoto, Kunio Hieshima, Seigo Sugiyama, Keizo Kajiwara, Katsunori Jinnouchi, Junji Saruwatari, Hideaki Jinnouchi

**Affiliations:** ^1^Division of Pharmacology and Therapeutics, Graduate School of Pharmaceutical Sciences, Kumamoto University, Kumamoto, Japan; ^2^Jinnouchi Hospital, Diabetes Care Center, Kumamoto, Japan

## Abstract

**Aim:** Sodium–glucose cotransporter 2 (SGLT2) inhibitors have been reported to increase hemoglobin levels; however, little is known about the magnitude of their anemia-improving effect in patients with advanced chronic kidney disease. We aimed to determine the influence of albuminuria and renal dysfunction on the anemia-improving effects of SGLT2 inhibitors in patients with Type 2 diabetes (T2D).

**Materials and Methods:** In this clinically based retrospective longitudinal study, the records of 4664 consecutive patients with T2D were reviewed. We investigated the effects of albuminuria and eGFR at baseline on change in hemoglobin after 3 months of SGLT2 inhibitor treatment by a multivariable linear regression analysis with calculation of the unstandardized partial regression coefficient (*B*).

**Results:** Among the patients with T2D, 230 patients (male, *n* = 170; female, *n* = 60; age, 67.0 ± 11.5 years) were eligible for the analysis. Patients with macroalbuminuria (urine albumin-to-creatinine ratio (uACR) > 300 mg/g or urine protein-to-creatinine ratio (uPCR) > 500 mg/g) exhibited a significantly smaller increase in hemoglobin after the initiation of SGLT2 inhibitor treatment (*B* −5.923 g/L, *p* = 0.003) compared to patients with normoalbuminuria (uACR < 30 mg/g or uPCR < 150 mg/g). Furthermore, almost all patients with proteinuria in the “nephrotic range,” defined as uPCR > 3500 mg/g, did not experience increased hemoglobin from SGLT2 inhibitor treatment. However, we could not find a significant association between the eGFR at baseline (including eGFR ≤ 15 mL/min/1.73 m^2^) and change in hemoglobin with SGLT2 inhibitor treatment.

**Conclusions:** Our findings indicate that severely increased albuminuria attenuates the anemia-improving effect of SGLT2 inhibitors for at least 3 months after their administration.

## 1. Introduction

Sodium–glucose cotransporter 2 (SGLT2) inhibitors have been reported to increase hemoglobin and hematocrit levels in patients with or without Type 2 diabetes (T2D) [1–5]. Increased hemoglobin or hematocrit levels are associated with increased oxygen delivery in circulating plasma, which may contribute to the prevention of cardiorenal events in patients treated with SGLT2 inhibitors [5, 6]. In the exploratory post hoc analysis of the EMPA-REG OUTCOME trial, changes in hematocrit and hemoglobin were the most important mediators of the reduced risk of cardiovascular death associated with empagliflozin compared to placebo [5]. These characteristics of SGLT2 inhibitors are completely different from erythropoiesis-stimulating agents (ESAs), which are commonly used to treat anemia in patients with chronic kidney disease (CKD) and increase the risk of mortality or cardiovascular events at high doses [7, 8]. Therefore, the effect of SGLT2 inhibitors on anemia has been emphasized in recent years.

As CKD progresses, the interstitial cells transform into myofibroblasts that can no longer synthesize erythropoietin (EPO), resulting in anemia [9]. While albuminuria is an indicator of the risk of developing or progressing to CKD or cardiovascular disease, its relationship to anemia has not been fully studied. In the post hoc analysis of the DAPA-CKD trial, dapagliflozin treatment was associated with increased hematocrit and anemia correction, irrespective of estimated glomerular filtration rate (eGFR) (≥ 45 or < 45 mL/min/1.73 m^2^) or urine albumin-to-creatinine ratio (uACR) (≤ 1000 or > 1000 mg/g) [2]. Eligible patients in this trial, however, had an eGFR of 25–75 mL/min/1.73m^2^ and a uACR of 200–5000 mg/g at baseline [2]. In addition, most of the patients enrolled in previous clinical trials of SGLT2 inhibitors did not have an eGFR of < 30 mL/min/1.73m^2^ [1–5]. To date, there have been no reports of a detailed investigation into the anemia-improving effect of SGLT2 inhibitors across a wide range of eGFR levels and albuminuria severities. Moreover, participants in clinical trials tend to be relatively stable and highly adherent, whereas real-world evidence remains lacking. In the present study, we investigated the influence of renal dysfunction and albuminuria on the anemia-improving effects of SGLT2 inhibitors in a clinical setting.

## 2. Materials and Methods

### 2.1. Participants and the Study Protocol

In this clinically based retrospective longitudinal study, the records of 4664 consecutive patients with T2D who visited Jinnouchi Hospital, Diabetes Care Center in Kumamoto, Japan, between January 2018 and September 2022 were reviewed. Among them, patients who were < 18 years of age; patients who had a history of dialysis, polycystic kidney disease, lupus nephritis, or antineutrophil cytoplasmic antibody-associated vasculitis; patients who were not on continuous SGLT2 inhibitor therapy during the evaluation period of anemia; and patients with incomplete data were excluded. This study was conducted in accordance with the Declaration of Helsinki, and the study protocol was approved by the Human Ethics Review Committee of Jinnouchi Hospital (No. 2023-1-1) and the Institutional Ethics Committee of the Faculty of Life Sciences, Kumamoto University (No.169). All participants provided their written informed consent before enrollment in the study.

### 2.2. Measurements and Definitions

Patient characteristics at the time of the first prescription of an SGLT2 inhibitor (0 months, baseline) and 3 months later were obtained from medical records. As hemoglobin or hematocrit seemed to be almost at a steady state at 2–4 months after starting SGLT2 inhibitor treatment [2, 5, 10, 11], the values at 3 months were used for the analyses. In cases where there was no visit at 3 months, data from up to 6 months later were used. T2D was defined according to the Japanese Clinical Practice Guidelines for Diabetes [12] or a patient history of T2D. Body mass index (BMI) was calculated by dividing the weight in kilograms by the square of height in meters. The eGFR was calculated using the Japanese eGFR-estimating equation, which has relatively little bias across the wide range of GFR values in the Japanese population: eGFR (mL/min/1.73 m^2^) = 194 × SCr^−1.094^ × Age^−0.287^ × 0.739 (if female) [13]. According to the Kidney Disease Improving Global Outcomes guidelines, the eGFR categories were defined as follows: G1 (normal or high), G2 (mildly decreased), G3a (mildly to moderately decreased), G3b (moderately to severely decreased), G4 (severely decreased), or G5 (kidney failure): ≥ 90, ≥ 60 and < 90, ≥ 45 and < 60, ≥ 30 and < 45, ≥ 15 and < 30, or < 15 mL/min/1.73 m^2^, respectively. Albuminuria is classified as A1 (normoalbuminuria), A2 (microalbuminuria), or A3 (macroalbuminuria) according to the uACR in an isolated urine sample for values < 30, 30–300, or > 300 mg/g, respectively [14]. In Japan, the urine protein-to-creatinine ratio (uPCR) is commonly used to classify the severity of CKD after the diagnosis of early-stage diabetic nephropathy in patients with diabetes, and a uPCR of 150–500 mg/g is considered equivalent to A2 albuminuria. Therefore, in the absence of urine albumin data, uPCR was used instead with < 150, 150–500, and > 500 mg/g [15].

### 2.3. Statistical Analyses

Data are presented as the mean ± standard deviation (SD) for continuous variables and as the number of individuals (percentage) for categorical variables. Group comparisons were performed using one-way analysis of variance for continuous variables and Fisher's exact test for categorical variables. Hemoglobin, hematocrit, the mean corpuscular volume (MCV), the mean corpuscular hemoglobin (MCH), and the mean corpuscular hemoglobin concentration (MCHC) at baseline and 3 months were compared using a paired *t*-test. The impact of albuminuria and eGFR on hemoglobin increase following SGLT2 inhibitor treatment was assessed using multivariate linear regression analysis, with unstandardized partial regression coefficient (*B*) calculations. Confounding factors were considered as follows: baseline characteristics, including age, sex, BMI, systolic blood pressure (SBP), and hemoglobin. Concomitant medications, such as iron preparations (oral tablets and/or injections), ESAs, angiotensin-converting enzyme (ACE) inhibitors, and/or angiotensin receptor blockers (ARBs) taken from baseline to just before the assessment at 3 months, were also considered as potential confounders. Hypoxia-inducible factor-prolyl hydroxylase (HIF-PH) inhibitors were not used in this study. Before incorporating these potential confounding factors into the linear regression model, the interactive effects of these factors on the increase in hemoglobin level with SGLT2 inhibitor treatment were analyzed using a two-way analysis of variance. When the interaction between the two factors was statistically significant, a multivariate linear regression analysis was performed separately for patients with and without either factor. Two-tailed *p* values of < 0.05 were considered statistically significant. Multiple comparisons were corrected using Bonferroni's method, and *p* values of <0.05/*n* were considered statistically significant after correcting for the number of comparisons. Statistical analyses were performed using SPSS software package for Windows (Version 24.0, IBM Japan Ltd., Tokyo, Japan).

## 3. Results

Among the 4664 patients with T2D, subjects were excluded in the following order: age < 18 years (*n* = 26), with a history of dialysis at the Jinnouchi Hospital (*n* = 21), with no continuous treatment with SGLT2 inhibitors during the evaluation period of anemia (*n* = 3469), and with incomplete data or a history of dialysis at another hospital (*n* = 918). Consequently, 230 subjects (170 males and 60 females, 67.0 ± 11.5 years) were included in the analysis. The study subjects had no history of polycystic kidney disease, lupus nephritis, or antineutrophil cytoplasmic antibody-associated vasculitis. The SGLT2 inhibitors prescribed to the patients included luseogliflozin (*n* = 86), empagliflozin (*n* = 52), dapagliflozin (*n* = 38), ipragliflozin (*n* = 35), tofogliflozin (*n* = 17), and canagliflozin (*n* = 2). The baseline characteristics of the study participants are presented in Table 1.

**Figure 1:**
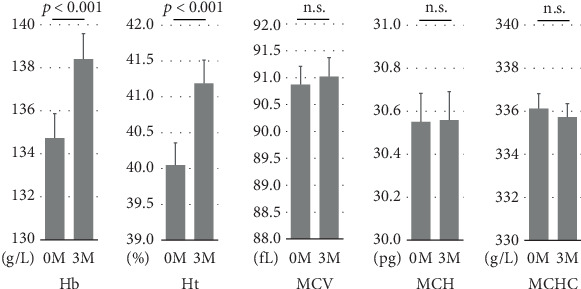
Changes in hemoglobin (Hb), hematocrit (Ht), mean corpuscular volume (MCV), mean corpuscular hemoglobin (MCH), and mean corpuscular hemoglobin concentration (MCHC) from baseline (0 M) to 3 months after SGLT2 inhibitor administration (3 M). n.s. not significant.

**Figure 2:**
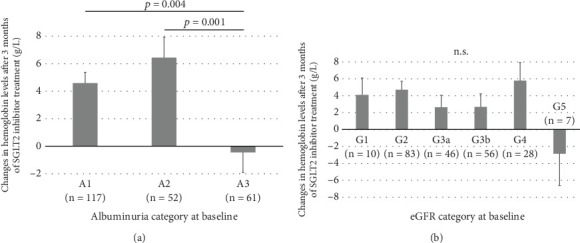
Changes in hemoglobin levels after 3 months of SGLT2 inhibitor treatment according to (a) albuminuria category and (b) eGFR category at baseline. Albuminuria categories were defined as A1 (uACR < 30 or uPCR < 150), A2 (uACR 30–300 or uPCR 150–500), or A3 (uACR > 300 or uPCR > 500). eGFR categories were defined as G1 (eGFR ≥ 90), G2 (eGFR ≥ 60 and < 90), G3a (eGFR ≥ 45 and < 60), G3b (eGFR ≥ 30 and < 45), G4 (eGFR ≥ 15 and < 30), or G5 (eGFR < 15). uACR, urine albumin-to-creatinine ratio (milligram/gram); uPCR, urine protein-to-creatinine ratio (milligram/gram); eGFR, estimated glomerular filtration rate (milliliter/minute/1.73 m^2^).

At 3 months, hemoglobin and hematocrit levels were significantly increased relative to baseline, while no significant changes were observed in MCV, MCH, and MCHC (Figure 1). The mean increases in hemoglobin and hematocrit levels after 3 months of SGLT2 inhibitor treatment were 3.7 g/L and 1.1%, respectively, with no marked differences between males and females. According to the albuminuria category at baseline, the mean increase in hemoglobin was significantly smaller in Group A3 than in Groups A1 or A2 (Figure 2a). There were no significant differences in the mean increase in hemoglobin between the eGFR categories at baseline (Figure 2b). Patients with A3 had significantly higher SBP and lower hemoglobin, eGFR, and serum iron levels at baseline than those with A1 or A2 (Table S1). Concomitant use of ESAs or ACE inhibitors/ARBs was observed more frequently in Group A3. The rate of concomitant iron use was 11.1% in Group A1, 15.4% in Group A2, and 9.8% in Group A3, with no significant difference between these groups (Table S1). The baseline characteristics of the participants according to eGFR categories are shown in Table S2. Multivariable linear regression analysis showed that patients with baseline A3 exhibited a significantly smaller increase in hemoglobin following the initiation of SGLT2 inhibitor treatment compared to those with baseline A1 (*B* −5.923, *p* = 0.003), even after adjustment for confounding factors (Table 2). Age and hemoglobin at baseline were significantly negatively associated with change in hemoglobin with SGLT2 inhibitor treatment (Table 2). Sex was not included in this linear regression analysis because there was a significant interaction between sex and concomitant ESA use (*p* = 0.017). Analyses stratified by sex also indicated that patients with baseline A3 showed a significantly smaller increase in hemoglobin after initiating SGLT2 inhibitor treatment compared to those with baseline A1 in both sexes (Table S3). Concomitant ESA use was strongly associated with change in hemoglobin with SGLT2 inhibitor treatment in females, whereas only six females were on ESAs and five of them had macroalbuminuria (Table S3).

**Figure 3:**
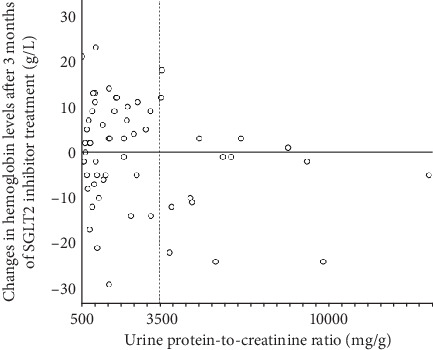
The correlation between the urine protein-to-creatinine ratio (milligram/gram) at baseline and changes in hemoglobin levels after 3 months of SGLT2 inhibitor treatment in patients with baseline A3.

Figure 3 shows the correlation between uPCR and change in hemoglobin after 3 months of SGLT2 inhibitor treatment in patients with baseline A3. Interestingly, almost all patients with nephrotic range proteinuria (uPCR > 3500 mg/g) had unchanged or decreased hemoglobin levels after the initiation of SGLT2 inhibitor treatment.

## 4. Discussion

This study is the first to elucidate the impact of albuminuria and renal dysfunction on the anemia-improving effects of SGLT2 inhibitors by analyzing real-world clinical data from patients exhibiting a spectrum of albuminuria severity and eGFR levels. We found that patients with macroalbuminuria exhibited a significantly smaller increase in hemoglobin levels after the initiation of SGLT2 inhibitor treatment than patients with normoalbuminuria. Additionally, almost all patients with proteinuria in the “nephrotic range,” as defined by a uPCR of > 3500 mg/g [16], showed no increase in hemoglobin with SGLT2 inhibitor treatment. On the other hand, eGFR at baseline was not significantly associated with changes in hemoglobin levels with SGLT2 inhibitor treatment.

The post hoc analyses of the DAPA-CKD and CREDENCE trials showed that both baseline albuminuria and eGFR had minimal impact on the hazard ratios (HRs) for anemia outcomes of dapagliflozin and canagliflozin relative to placebo [1, 2]. In these analyses, participants were stratified into uACR-based groups (≤ 1000 vs. > 1000 mg/g in DAPA-CKD and < 1000 vs. ≥ 1000 mg/g in CREDENCE), and the effects of dapagliflozin and canagliflozin on anemia outcomes were assessed relative to the placebo within each subgroup. Notably, the inclusion criteria for DAPA-CKD encompassed a uACR range of 200–5000 mg/g, and for CREDENCE, the criteria required a uACR > 300–≤5000 mg/g. Therefore, in these subgroup analyses, patients with relatively severe albuminuria (i.e., 200–1000 mg/g in DAPA-CKD and > 300–< 1000 mg/g in CREDENCE) and those with more severe albuminuria were compared. In contrast, in this study, patients with normoalbuminuria served as the reference group, and comparisons were made between patients with normoalbuminuria and those with microalbuminuria, as well as between patients with normoalbuminuria and those with macroalbuminuria. We speculate that this difference in reference criteria may have contributed to the limited identification of the influence of albuminuria on the effects of dapagliflozin and canagliflozin on anemia outcomes within the subgroup analyses of DAPA-CKD and CREDENCE. Incidentally, post hoc analyses of these trials have demonstrated a long-term anemia-improving effect of dapagliflozin and canagliflozin in populations primarily composed of individuals with “macroalbuminuria.” However, caution is warranted when interpreting these findings as the primary outcome differs from that of the present study. Fundamentally, these trials were not designed to elucidate the impact of albuminuria or renal dysfunction on the anemia-improving effects of SGLT2 inhibitors. Additionally, in both trials, appropriate addition of anemia treatments, including iron supplementation, was permitted. In contrast, in this study, the frequency of iron supplementation up to the time of anemia evaluation (i.e., 3 months later) was found to be very low at < 10% in patients with macroalbuminuria. The possibility that the underlying iron deficiency contributed to the attenuation of the anemia-improving effect of SGLT2 inhibitors cannot be ruled out. Nevertheless, while the average increase in hemoglobin level was significantly smaller in patients with macroalbuminuria than in those with normoalbuminuria, individual variability in this change was still observed among patients with macroalbuminuria (Figure 3). In the nephrotic range proteinuria, however, the hemoglobin increase was almost consistently absent.

The effect of SGLT2 inhibitors on hemoglobin and hematocrit increases has been suggested to be due to hemoconcentration resulting from glucosuria and natriuresis [17]. However, the diuretic effects of SGLT2 inhibitors are evident very early in the course of treatment (< 1 week). In contrast, the increase in hemoglobin and hematocrit emerges after the first week of SGLT2 inhibitor treatment and peaks after 2–4 months [2, 5, 10, 11]. Instead of hemoconcentration, it is proposed that SGLT2 inhibitors increase hemoglobin and hematocrit by stimulating EPO production [9, 18]. In addition, the ability of SGLT2 inhibitors to improve iron mobilization by derepressing hepcidin and ferritin would be expected to increase cytosolic bioreactive iron [9]. These effects would allow SGLT2 inhibitors to facilitate erythropoiesis in the bone marrow [9, 18].

Studies in both humans and animals have reported substantial urinary losses of EPO in nephrotic syndrome [19, 20]. Although urinary EPO losses do not necessarily appear to be accompanied by a decrease in adequate plasma concentrations of EPO, urinary losses have been shown to contribute to EPO deficiency anemia [19–22]. Zhou and Vaziri examined the changes in plasma EPO concentration and urinary EPO excretion in response to hypobaric hypoxia and experimentally induced anemia via phlebotomy and volume replacement in nephrotic and control rats [20]. Both hypoxia and anemia experiments demonstrated a robust increase in plasma EPO concentration with no detectable urinary EPO excretion in control rats. In contrast, nephrotic rats exhibited a negligible increase in plasma EPO concentration accompanied by a substantial increase in urinary EPO excretion. These results suggest that significant urinary EPO excretion in nephrotic animals plays a major role in the development of relative EPO deficiency [20].

Similarly, a marked increase in the urinary excretion of transferrin and iron has been observed in patients with nephrosis. In that study, serum iron concentrations gradually decreased with increasing albuminuria and urinary transferrin and iron [23]. A gradual decrease in serum iron concentration with increasing albuminuria was also observed in the present study. It has been suggested that although urinary iron loss in patients with nephrotic syndrome does not usually result in severe iron deficiency, it may decrease iron stores and increase the risk of developing incidental iron deficiency anemia [21, 23]. Given the above, the attenuated or diminished anemia-improving effect of SGLT2 inhibitors in this study in patients with nephrotic range proteinuria may be due to the urinary losses of EPO, transferrin, and/or iron, which are considered to be crucial for the promotion of erythropoiesis by SGLT2 inhibitors.

Additionally, inflammation may be a potential mechanism underlying the impact of macroalbuminuria on the anemia-improving effects of SGLT2 inhibitors. SGLT2 inhibitors have been reported to exert both direct and indirect anti-inflammatory effects, with the latter driven by the regulation of various metabolic pathways [24, 25]. These anti-inflammatory effects are considered one of the key mechanisms underlying the downstream improvement in iron mobilization and enhancement of EPO production [9]. On the other hand, several inflammatory markers have been reported to be elevated in patients with macroalbuminuria or overt nephropathy [26, 27]. Therefore, severely increased albuminuria may adversely affect EPO production and iron utilization through inflammation, potentially attenuating the anemia-improving effect of SGLT2 inhibitors. In the absence of data on the urinary losses of EPO, transferrin, iron, and inflammatory markers in this study, the above mechanisms are speculative but considered logically plausible. Further investigation, including the urinary loss of these substances and metrics of inflammation in humans, is therefore warranted to elucidate the underlying mechanisms of the possible attenuated or diminished anemia-improving effect of SGLT2 inhibitors in patients with severely increased albuminuria.

In the present study, we could not find a significant association between eGFR at baseline, including ≤ 15 mL/min/1.73 m^2^, and change in hemoglobin with SGLT2 inhibitor treatment. SGLT2 inhibition has been proposed to stimulate EPO production through mechanisms involving both the kidneys and the liver. While the kidneys are the primary regulators in adults, the liver—initially the main site of EPO synthesis during fetal development—continues to contribute partially to its production in adulthood [9]. Advanced renal dysfunction *per se* may not be fatal to the anemia-improving effect of SGLT2 inhibitors. In contrast, a retrospective cohort study previously reported that eGFR ≤ 15 mL/min/1.73 m^2^ was associated with a significantly lower incidence of > 3.0 g/L increase in hemoglobin at 6 months after the initiation of SGLT2 inhibitor treatment in comparison to eGFR > 60 mL/min/1.73 m^2^ [28]. Because of the small sample size in this and previous studies, further research is needed to determine the association between advanced renal dysfunction and the effect of SGLT2 inhibitors on anemia.

In this study, the mean increase in hemoglobin after 3 months of treatment with SGLT2 inhibitors was 3.7 g/L (134.7 g/L at baseline, 138.4 g/L at 3 months). A post hoc analysis of the CREDENCE trial showed a comparable increase in hemoglobin (approximately 4 g/L) after 12 months of treatment with canagliflozin in T2D patients whose mean hemoglobin concentration at baseline was 132.6 g/L. In the same study, the canagliflozin group had a lower incidence of composite anemia events (HR, 0.65; 95% confidence interval (CI), 0.55–0.77), anemia events alone (HR, 0.58; 95% CI, 0.47–0.72), and need for ESAs (HR, 0.65; 95% CI, 0.46–0.91) than the placebo group during a median follow-up of 2.6 years [1]. These results suggest that even a modest increase in hemoglobin levels with SGLT2 inhibitors can reduce the development of anemia and the need for ESAs. Although we could not evaluate the impact of SGLT2 inhibitors on the incidence of anemia events or the need for ESAs due to the nonplacebo-controlled setting, we believe that the change in hemoglobin level with SGLT2 inhibitor treatment observed in this study was clinically significant. On the other hand, almost all patients with nephrotic range proteinuria had no increase in hemoglobin with SGLT2 inhibitor treatment in this study, suggesting that the expected anemia-improving effect of SGLT2 inhibitors may not be observed depending on the severity of albuminuria.

Furthermore, we showed that the hemoglobin level at baseline was negatively associated with change in hemoglobin after initiation of SGLT2 inhibitor treatment. This is consistent with previous reports that, in the SGLT2 inhibitor-treated group, the increase in hemoglobin from baseline was smaller in participants with higher hemoglobin levels at baseline and in those who were not anemic than in others [3, 4]. The mean increase in hemoglobin with SGLT2 inhibitors in A1 patients in this study was smaller than that in A2 patients, possibly because of their higher hemoglobin levels at baseline.

In the present study, the age at baseline was significantly negatively associated with change in hemoglobin level with SGLT2 inhibitor treatment. The exact mechanisms underlying this relationship remain unknown. In contrast, anemia in the elderly is well known to be associated with malnutrition, such as iron deficiency, and/or chronic disease and inflammation, with inhibited production and action of EPO and increased hepcidin [29]. Therefore, elderly patients with anemia are likely to be deficient in iron and EPO, which are essential for the anemia-improving effects of SGLT2 inhibitors, and the treatment-induced increase in hemoglobin tends to be smaller than that in nonelderly patients. On the other hand, a significant interactive effect between female sex and concomitant ESA use on the increase in hemoglobin with SGLT2 inhibitor treatment was found in this study. However, only six females were on ESAs, and five of them had macroalbuminuria. Although sex differences in the effects of medication are important issues, the sample size in this study was too small to determine the sex-specific effect of ESAs on the increase in hemoglobin with SGLT2 inhibitor treatment.

The present study was associated with several limitations. First, it was observational in order to best reflect the actual clinical situation; however, it was a retrospective study that was conducted in a single center. Second, we could not compare the effects of SGLT2 inhibitors to a placebo. Thus, it was not possible to determine the quantitative magnitude of the influence of severely increased albuminuria on the hemoglobin-increasing effect of SGLT2 inhibitors while accounting for variations in hemoglobin due to its natural course. Third, the long-term effects of SGLT2 inhibitors on anemia have not been studied. Finally, we did not obtain sufficient data on ferritin levels (only 15.7% of participants), transferrin saturation, serum concentrations of EPO and hepcidin, or other metrics of inflammation that might have confounded the interpretation of our results. In particular, it should be noted that there were no data on the urinary loss of EPO, iron, or transferrin, which are essential to substantiate the mechanisms we have discussed.

## 5. Conclusion

The results of the present study suggest that severely increased albuminuria may attenuate the anemia-improving effect of SGLT2 inhibitors for at least 3 months after their initiation. Given the cardio-renoprotective effects of SGLT2 inhibitors, the addition of SGLT2 inhibitors while reducing ESA requirements is expected to reduce the risk of cardiovascular complications in high-risk patients, while achieving good anemia control. We hope that the results of this study will help to predict the ineffectiveness of SGLT2 inhibitors in advance and contribute to more effective pharmacotherapy for anemia in CKD patients with diabetes. Further large-scale prospective investigations, including the analysis of longitudinal and quantitative data on albuminuria and other laboratory/descriptive information, are needed to validate the present findings.

## Figures and Tables

**Table 1:** Baseline characteristics of the participants.

	**n** = 230
Age (years)	67.0 ± 11.5
Female sex	60 (26.1)
BMI (kg/m^2^)	25.3 ± 3.9
Systolic blood pressure (mmHg)	134.0 ± 15.3
Diastolic blood pressure (mmHg)	72.9 ± 11.2
HbA1c (%)	7.5 ± 1.4
HbA1c (mmol/mol)	58.3 ± 15.8
Hemoglobin (g/L)	134.7 ± 17.3
Hematocrit (%)	40.0 ± 4.7
MCV (fL)	90.9 ± 5.1
MCH (pg)	30.6 ± 2.0
MCHC (g/L)	336.1 ± 10.7
Albuminuria	
A1	117 (50.9)
A2	52 (22.6)
A3	61 (26.5)
eGFR (mL/min/1.73 m^2^)	53.6 ± 22.0
G1 (eGFR ≥ 90)	10 (4.3)
G2 (eGFR ≥ 60 and < 90)	83 (36.1)
G3a (eGFR ≥ 45 and < 60)	46 (20.0)
G3b (eGFR ≥ 30 and < 45)	56 (24.3)
G4 (eGFR ≥ 15 and < 30)	28 (12.2)
G5 (eGFR < 15)	7 (3.0)
Serum iron (*μ*g/dL)	85.8 ± 33.4
Concomitant medications^a^	
Iron preparations^b^	27 (11.7)
ESAs	40 (17.4)
ACE inhibitors/ARBs	128 (55.7)

*Note:* Data are presented as the number (percentage) and mean ± SD. Albuminuria was defined as A1 (uACR < 30 mg/g or uPCR < 150 mg/g), A2 (uACR 30–300 mg/g or uPCR 150–500 mg/g), or A3 (uACR > 300 mg/g or uPCR > 500 mg/g).

Abbreviations: ACE, angiotensin-converting enzyme; ARB, angiotensin receptor blocker; BMI, body mass index; eGFR, estimated glomerular filtration rate; ESA, erythropoietin-stimulating agent; HbA1c, glycated hemoglobin; MCH, mean corpuscular hemoglobin; MCHC, mean corpuscular hemoglobin concentration; MCV, mean corpuscular volume; SD, standard deviation; uACR, urine albumin-to-creatinine ratio; uPCR, urine protein-to-creatinine ratio.

^a^Concomitant medications taken from baseline to just before the assessment at 3 months.

^b^Oral tablets and/or injections.

**Table 2:** Baseline variables associated with increased hemoglobin after 3 months of SGLT2 inhibitor treatment.

	**B**	**SE**	**p** ** value**
Albuminuria			
A1	Ref.	—	—
A2	0.957	1.670	0.567
A3	**−5.923**	**1.954**	**0.003**
eGFR			
G1 (eGFR ≥ 90)	Ref.	—	—
G2 (eGFR ≥ 60 and < 90)	0.669	3.152	0.832
G3a (eGFR ≥ 45 and < 60)	−0.582	3.374	0.863
G3b (eGFR ≥ 30 and < 45)	−0.406	3.527	0.908
G4 (eGFR ≥ 15 and < 30)	2.609	4.012	0.516
G5 (eGFR < 15)	−5.090	5.164	0.325
Age (/year)	−**0.156**	**0.067**	**0.020**
BMI (/kg/m^2^)	0.059	0.171	0.733
Systolic blood pressure (/mmHg)	−0.070	0.044	0.115
Hemoglobin (/g/L)	−**0.238**	**0.049**	**< 0.001**
Iron preparations^a,b^	1.934	2.054	0.348
ESAs^a^	−3.275	2.173	0.133
ACE inhibitors/ARBs^a^	0.403	1.350	0.766

*Note:* The bold values indicate variables with a significant association with increased hemoglobin with SGLT2 inhibitor treatment.

Abbreviations: *B*, unstandardized partial regression coefficient; SE, standard error.

^a^Concomitant medications taken from baseline to just before the assessment at 3 months.

^b^Oral tablets and/or injections.

## Data Availability

The data that support the findings of this study are available from the corresponding authors upon reasonable request.

## Supporting Information

Supporting Information Additional supporting information can be found online in the Supporting Information section. Table S1. Baseline characteristics of the participants according to albuminuria categories. Table S2. Baseline characteristics of the participants according to eGFR categories. Table S3. Baseline variables associated with increased hemoglobin after 3 months of SGLT2 inhibitor treatment by sex.
