# Supplementary material for: Impact of Albuminuria and Renal Dysfunction on the Anemia-Improving Effect of SGLT2 Inhibitors in Patients With Type 2 Diabetes: A Real-World Observational Study
Source: J Diabetes Res. 2025 Jun 28;2025:5399360. doi: 10.1155/jdr/5399360 (PMC12228570; doi:10.1155/jdr/5399360)
Supplement: Supporting Information — Additional supporting information can be found online in the Supporting Information section. Table S1. Baseline characteristics of the participants according to albuminuria categories. Table S2. Baseline characteristics of the participants according to eGFR categories. Table S3. Baseline variables associated with increased hemoglobin after 3 months of SGLT2 inhibitor treatment by sex. [file 5399360.f1.docx]

Supplemental Table 1. Baseline characteristics of the participants according to albuminuria categories

|  | A1  (n=117) | A2  (n=52) | A3  (n=61) | P-value |
| --- | --- | --- | --- | --- |
| Age (years) | 65.3 ± 11.6 | 68.6 ± 12.0 | 68.6 ± 10.4 | 0.084 |
| Female sex (%) | 35 (29.9) | 12 (23.1) | 13 (21.3) | 0.435 |
| Body mass index (kg/m^2^) | 25.2 ± 3.8 | 26.0 ± 4.5 | 25.0 ± 3.5 | 0.303 |
| Systolic blood pressure (mmHg) | **129.7 ± 13.5** | **135.8 ± 15.5^*^** | **140.8 ± 15.9^*^** | **<0.001** |
| Diastolic blood pressure (mmHg) | 73.1 ± 10.2 | 72.0 ± 10.8 | 73.2 ± 13.4 | 0.809 |
| HbA1c (%) | 7.5 ± 1.5 | 7.4 ± 1.1 | 7.3 ± 1.5 | 0.557 |
| HbA1c (mmol/mol) | 59.4 ± 16.6 | 57.8 ± 12.3 | 56.7 ± 16.8 | 0.557 |
| Hemoglobin (g/L) | **140.1 ± 15.7** | **132.1 ± 16.8^*^** | **126.6 ± 17.2^*^** | **<0.001** |
| Hematocrit (%) | **41.5 ± 4.2** | **39.5 ± 4.6^*^** | **37.8 ± 4.8^*^** | **<0.001** |
| MCV (fL) | 91.2 ± 4.3 | 91.4 ± 5.2 | 89.9 ±6.4 | 0.204 |
| MCH (pg) | 30.8 ± 1.7 | 30.5 ± 2.0 | 30.1 ± 2.4 | 0.093 |
| MCHC (g/L) | 337.7 ± 10.5 | 334.1 ± 10.5 | 334.8 ± 11.1 | 0.075 |
| eGFR (mL/min/1.73m^2^) | **63.7 ± 17.6** | **52.0 ± 21.7^*^** | **35.5 ± 17.7^*,**^** | **<0.001** |
| G1 (eGFR ≥90) | 9 (7.7) | 1 (1.9) | 0 (0.0) | N.A. |
| G2 (eGFR ≥60 and <90) | 60 (51.3) | 16 (30.8) | 7 (11.5) |  |
| G3a (eGFR ≥45 and <60) | 27 (23.1) | 12 (23.1) | 7 (11.5) |  |
| G3b (eGFR ≥30 and <45) | 19 (16.2) | 15 (28.8) | 22 (36.1) |  |
| G4 (eGFR ≥15 and <30) | 2 (1.7) | 7 (13.5) | 19 (31.1) |  |
| G5 (eGFR <15) | 0 (0.0) | 1 (1.9) | 6 (9.8) |  |
| Concomitant medications^†^ |  |  |  |  |
| Iron preparations^‡^ | 13 (11.1) | 8 (15.4) | 6 (9.8) | 0.663 |
| ESAs | **7 (6.0)** | **8 (15.4)** | **25 (41.0)^*,**^** | **<0.001** |
| ACE inhibitors/ARBs | **45 (38.5)** | **34 (65.4)^*^** | **49 (80.3)^*^** | **<0.001** |
| Serum iron (µg/dL) | **92.5 ± 37.6** | **81.7 ± 29.3** | **76.7 ± 24.8^*^** | **0.006** |

Continuous variables were compared using a one-way analysis of variance; the categorical variables were compared using Fisher’s exact test. Multiple comparisons were corrected using the Bonferroni method. Albuminuria was defined as A1 (uACR <30 mg/g or uPCR <150 mg/g), A2 (uACR 30–300 mg/g or uPCR 150–500 mg/g) or A3 (uACR >300 mg/g or uPCR >500 mg/g).

^*^<0.05 vs. A1; ^**^<0.05 vs. A2; ^†^Concomitant medications taken from baseline to just before the assessment at 3 months; ^‡^Oral tablets and/or injections.

ACE, angiotensin-converting enzyme; ARB, angiotensin receptor blocker; BMI, body mass index; eGFR, estimated glomerular filtration rate; ESA, erythropoietin stimulating agent; HbA1c, glycated hemoglobin; MCH, mean corpuscular hemoglobin; MCHC, mean corpuscular hemoglobin concentration; MCV, mean corpuscular volume; N.A., not available; uACR, urine albumin-to-creatinine ratio; uPCR, urine protein-to-creatinine ratio.

Supplemental Table 2. Baseline characteristics of the participants according to eGFR categories

|  | G1  (n=10) | G2  (n=83) | G3a  (n=46) | G3b  (n=56) | G4  (n=28) | G5  (n=7) |
| --- | --- | --- | --- | --- | --- | --- |
| Age (years) | 54.2 ± 13.8 | 62.4 ± 11.5 | 68.7 ± 8.7 | 72.7 ± 9.2 | 71.2 ± 9.8 | 65.7 ± 13.6 |
| Female sex (%) | 3 (30.0) | 25 (30.1) | 12 (26.1) | 11 (19.6) | 8 (28.6) | 1 (14.3) |
| Body mass index (kg/m^2^) | 24.8 ± 5.1 | 26.0 ± 4.4 | 24.9 ± 3.6 | 25.2 ± 3.6 | 24.6 ± 3.2 | 24.6 ± 3.9 |
| Systolic blood pressure (mmHg) | 131.1 ± 16.6 | 134.7 ± 14.4 | 132.4 ± 16.1 | 133.1 ± 15.3 | 135.4 ± 16.9 | 142.3 ± 12.9 |
| Diastolic blood pressure (mmHg) | 77.1 ± 12.5 | 76.0 ± 9.5 | 73.9 ± 10.7 | 68.1 ± 9.9 | 70.0 ± 15.0 | 73.4 ± 11.7 |
| HbA1c (%) | 8.5 ± 2.0 | 7.6 ± 1.5 | 7.5 ± 1.1 | 7.6 ± 1.6 | 6.8 ± 1.0 | 6.4 ± 0.6 |
| HbA1c (mmol/mol) | 68.9 ± 22.3 | 59.8 ± 16.0 | 58.6 ± 12.6 | 59.5 ± 17.3 | 50.5 ± 11.2 | 46.1 ± 6.7 |
| Hemoglobin (g/L) | 148.2 ± 7.7 | 141.8 ± 14.5 | 139.4 ± 17.0 | 129.1 ± 14.8 | 117.7 ± 13.2 | 113.3 ± 10.3 |
| Hematocrit (%) | 43.7 ± 2.0 | 42.0 ± 3.8 | 41.5 ± 4.6 | 38.4 ± 4.2 | 35.4 ± 3.7 | 34.4 ± 3.0 |
| MCV (fL) | 92.0 ± 4.1 | 89.5 ± 5.3 | 91.9 ± 4.4 | 91.8 ± 5.3 | 91.3 ± 5.3 | 88.7 ± 4.7 |
| MCH (pg) | 31.3 ± 1.7 | 30.3 ± 2.3 | 30.9 ±1.7 | 30.8 ± 1.7 | 30.4 ± 2.0 | 29.3 ± 1.9 |
| MCHC (g/L) | 339.5 ± 8.1 | 337.6 ± 11.7 | 335.8 ± 10.7 | 336.1 ± 9.9 | 332.6 ± 10.0 | 329.4 ± 6.5 |
| Albuminueia |  |  |  |  |  |  |
| A1 | 9 (90.0) | 60 (72.3) | 27 (58.7) | 19 (33.9) | 2 (7.1) | 0 (0.0) |
| A2 | 1 (10.0) | 16 (19.3) | 12 (26.1) | 15 (26.8) | 7 (25.0) | 1 (14.3) |
| A3 | 0 (0.0) | 7 (8.4) | 7 (15.2) | 22 (39.3) | 19 (67.9) | 6 (85.7) |
| Concomitant medications^†^ |  |  |  |  |  |  |
| Iron preparations^‡^ | 0 (0.0) | 9 (10.8) | 4 (8.7) | 7 (12.5) | 7 (25.0) | 0 (0.0) |
| ESAs | 0 (0.0) | 0 (0.0) | 3 (6.5) | 14 (25.0) | 17 (60.7) | 6 (85.7) |
| ACE inhibitors/ARBs | 2 (20.0) | 37 (44.6) | 27 (58.7) | 38 (67.9) | 18 (64.3) | 6 (85.7) |
| Serum iron (µg/dL) | 101.9 ± 20.5 | 88.0 ± 32.5 | 88.2 ± 27.9 | 87.2 ± 42.8 | 68.2 ± 23.9 | 80.9 ± 19.1 |

eGFR categories were defined as G1 (eGFR ≥90), G2 (eGFR ≥60 and <90), G3a (eGFR ≥45 and <60), G3b (eGFR ≥30 and <45), G4 (eGFR ≥15 and <30), or G5 (eGFR <15). Albuminuria categories were defined as A1 (uACR <30 mg/g or uPCR <150 mg/g), A2 (uACR 30–300 mg/g or uPCR 150–500 mg/g) or A3 (uACR >300 mg/g or uPCR >500 mg/g).

^†^Concomitant medications taken from baseline to just before the assessment at 3 months; ^‡^Oral tablets and/or injections.

ACE, angiotensin-converting enzyme; ARB, angiotensin receptor blocker; BMI, body mass index; eGFR, estimated glomerular filtration rate; ESA, erythropoietin stimulating agent; HbA1c, glycated hemoglobin; MCH, mean corpuscular hemoglobin; MCHC, mean corpuscular hemoglobin concentration; MCV, mean corpuscular volume; uACR, urine albumin-to-creatinine ratio; uPCR, urine protein-to-creatinine ratio.

Supplemental Table 3. Baseline variables associated with increased hemoglobin after 3 months of SGLT2 inhibitor treatment by sex

|  | Males (n=170) | | |  | Females (n=60) | | |
| --- | --- | --- | --- | --- | --- | --- | --- |
|  | B | SE | *P*-value |  | B | SE | *P*-value |
| Albuminuria |  |  |  |  |  |  |  |
| A1 | Ref. | - | - |  | Ref. | - | - |
| A2 | 1.078 | 1.992 | 0.589 |  | -1.603 | 2.858 | 0.578 |
| A3 | **-6.533** | **2.374** | **0.007** |  | **-7.236** | **3.496** | **0.044** |
| eGFR |  |  |  |  |  |  |  |
| G1 (eGFR ≥90) | Ref. | - | - |  | Ref. | - | - |
| G2 (eGFR ≥60 and <90) | 2.913 | 3.808 | 0.446 |  | -4.519 | 4.949 | 0.366 |
| G3a (eGFR ≥45 and <60) | 1.945 | 4.041 | 0.631 |  | -6.936 | 5.410 | 0.206 |
| G3b (eGFR ≥30 and <45) | 2.249 | 4.264 | 0.599 |  | -7.070 | 5.788 | 0.228 |
| G4 (eGFR ≥15 and <30) | 5.658 | 4.862 | 0.246 |  | -2.434 | 6.640 | 0.716 |
| G5 (eGFR <15) | -4.917 | 5.932 | 0.408 |  | -4.303 | 10.881 | 0.694 |
| Age (/year) | **-0.261** | **0.083** | **0.002** |  | 0.068 | 0.112 | 0.550 |
| BMI (/kg/m^2^) | 0.206 | 0.231 | 0.373 |  | 0.228 | 0.232 | 0.332 |
| Systolic blood pressure (/mmHg) | -0.064 | 0.055 | 0.240 |  | -0.053 | 0.068 | 0.442 |
| Hemoglobin (/g/L) | **-0.322** | **0.059** | **<0.001** |  | -0.180 | 0.114 | 0.123 |
| Iron preparations^†, ‡^ | 4.218 | 2.582 | 0.104 |  | -0.706 | 3.203 | 0.826 |
| ESAs^†^ | -4.099 | 2.634 | 0.122 |  | **-10.061** | **4.575** | **0.033** |
| ACE inhibitors/ARBs^†^ | 0.139 | 1.647 | 0.933 |  | 1.307 | 2.489 | 0.602 |

The bold values indicate variables with a significant association with increased hemoglobin with SGLT2 inhibitor treatment.

^†^Concomitant medications taken from baseline to just before the assessment at 3 months; ^‡^Oral tablets and/or injections.

B, unstandardized partial regression coefficient; SE, standard error.
